# On Albumen in the Treatment of Pulmonary Consumption

**Published:** 1877-06

**Authors:** E. L. Shurly

**Affiliations:** Detroit, Mich.


BUFFALO

### Medical and Surgical Journal.

VOL XVI. JUNE, 1877. No. 11.

### Original Communications.

———:o:———

ART. I.—*On Albumen in the Treatment of Pulmonary Consumption.* By E. L. SHURLY, M. D., Detroit, Mich.

Written for the Buffalo Medical and Surgical Journal.

A disease so universally prevalent and disastrous among civilized nations as "Pulmonary Consumption" and one, too, which has thus far so successfully baffled the efforts of medical art in arresting its progress, must necessarily be the subject of much interest, thought and investigation, and encourage us to more vigorous pursuit after its mysterious operations. It must be conceded, that pathological investigators,—although deserving the highest praise and encouragement,—have as yet, only given us *relative* results—and nothing *absolute* as a substructure upon which to establish a rational plan of treatment for this disease; for, whether we regard phthisis as a vitiated neoplasm, an inflammation, or other of the supposed pathological processes, we are still "in the dark ' regarding the selection of therapeutic measures. Hence we are compelled to turn aside from this conflict of opinions and experiment, to place reliance more on the deductions from physiological, chemical and clinical data!

The following remarks in support of the plan of treatment to be suggested are almost entirely based upon the well known physiological and chemical processes connected with nutrition, processes, which to my mind, deserve the greatest consideration in the treatment, not only of this, but of all diseases.

In the first place, taking a summary physiological retrospect, we see that the animal body is not only constructed and repaired, but its living force maintained through material derived from without, called food; which by certain regular metamerphoses becomes converted to the use of the economy in sustaining the normal condition known as health; and further, that without a certain supply of this material, food, both in proper quantity and quality, the harmonious equilibrium of molecular action becomes disturbed, disease inevitably following, tending subsequently to decay.

Therefore, as living force cannot exist without material, and as "living force can generate the same amount of work as that expended in its production" (Helmholtz), it follows that a deprivation of food (whether from paucity of crude material or malassimilation) must cause a consumption of the already formed structures, so long as the living force continues its action within the body. So that, the *introduction* of the proper material for the play of life force becomes as important a factor of animal existence as the *operation* of the life force itself. Hence, it is obvious that our attention should be *concentrated* on the functions of digestion and assimilation on the one hand, and the character of the nutriment introduced on the other, while treating any wasting disease. Regarding the character of the food, physiological chemistry teaches with tolerable certainty that the four classes of elements, nitrogenous, hydro-carbons, carbo-hydrates, and inorganic, must each be represented in the food requisite for normal nutrition.

The carbo-hydrates and hydro-carbons; such as, fats, sugar and starch, are undoubtedly very important as *force producing agents*, for, as Pavy asserts, "That energy capable of resulting in the performance of mechanical work is produced in the animal system by the oxidation of carbonaceous matter may be considered an establisbed fact." Besides this, we have in support of the fact, the interesting experiments of Frankland, which prove by the number of units of heat evolved in the consumption of one gramme of each of the articles of these classes that fats take the first rank as force producing agents, thus displacing the doctrine promulgated by Liebig and for a long time accepted by the profession, that energy was especially the resultant of the direct oxidation of nitrogenous matter. The starchy and saccharine elements rank next in the tables of Frankland as force producing agents, while albumen ranks last. But, regarding the utility of these latter agents in this connection, it must be remembered that they both contribute by metamerphosis very largely to the amount of carbonaceous matter actually converted into force through oxidation, as proven by actual experiment on man and the lower animals, so that vital force, or work is by no means so dependant upon the amount of fat introduced from without as at first thought might appear.

Of course, each of the inorganic constituents of food has its particular office in the economy, but probably none are quite as useful as phosphorus, in its compounds, for it is a component of all the tissues; besides, by its presence serving an important purpose in the process of digestion. But of all the proximate principles the nitrogenous, and especially the albuminous, must be supplied in the most unvarying quantity, particularly as they are not produced within the body from a metamorphosis of either of the other classes; and regarding their importance, Lehman says, "If we pause for a moment to contemplate the great series of the chemical substrata of the animal body, we at once perceive that there are four principal groups of substances in which the vital processes are manifested with the greatest intensity. Amongst these the *albuminous substances* or the so-called protein-bodies and their *derivatives* are the most conspicuous. A mere superficial glance at the occurrence of albumen is sufficient to show that this must be one of the most important substances in the whole animal body; we have met with it in the largest quantity in the blood and in all those animal juices which contribute directly towards the nutrition of the organs, and a more careful examination of many of the animal tissues shows that albumen requires only some slight modifications to become consolidated under different forms; as, for instance, when it contributes towards the formation of the solid contractile parts, under the form of syntonin (musclefibrin), by which alone both the voluntary and involuntary movements of the animal body are effected.

We found it both in a dissolved and an undissolved form in the most delicate organic combinations, as, for instance, in the contents of the nerve tubes, structures by which the animal essentially differs from the plant, and in which the highest force of all animal life may be said to be located," and in another place he says of albumen: "Albumen occurs in all those animal substances which supply the whole body, or individual parts of it, with the materials necessary for nutrition and the renovation of effete matters. Hence, albumen is a principal constituent of the blood, the lymph, and chyle, as well as of all serous fluids."

Thus, taking the foregoing quotations (which, it may be added, can be corroborated by many others of equal authority), from Lehman, we see albumen standing forth the most important of all, as an element of nutrition. It is this in fact that constitutes protoplasm, the germ of animal as well as vegetable life, from the Bathybius, a mere mass of jelly through the nonnuclear and nuclear or higher cell, to the most complex organism. It stands as the quintessence of cell life, and the cell is but the being in miniature. Being thus conspicuously concerned in all physiological vital actions it must necessarily be abnormally destroyed or perverted by disease processes, which involve perhaps even greater molecular action than the normal vital processes; and this seems to be proven moreover, by various analyses of the products of inflammation, fatty, tuberculous and scrofulous degenerations, showing them to be very rich in albumen, especially pus, which according to Frey is only a dead cell (a cell at rest).

Now how much of a certain disease action depends upon the misdirection, so to speak, of the vital force itself, without reference to the quantity or character of the nutriment, of course, one cannot say, as living force is only known to us by its work and cannot be bridled and directed as electricity can. Nevertheless, if we know that its proper operation largely depends upon a supply of a certain proximate principle, as for instance albumen, and if we can then devise means of controlling or securing the introduction of this material to the system, it seems fair to infer that we shall to a certain extent be able to control the disease action itself. Thus in a wasting disease like Phthisis Pulmonalis, for instance, we must endeavor to introduce *more* than an ordinary supply of the proximate principles, especially the nitrogenous (which undoubtedly suffer the greatest destruction), that this rampant vital action may not consume the already formed and appropriated material; in other words, we must in some way supply the extra demand. But, it may be asked, how can we do this, in cases of this kind, where from want of appetite, from feeble digestion and assimilation, (almost constant accompaniments of Phthisis) we can scarcely introduce an amount of ordinary rich food requisite for healthy vital action, without producing indigestion with its reactionary disturbances. Shall we resort to tonics, or stomachics, in order to spur the feeble digestive apparatus? Not altogether, for common experience teaches that many of these cases, and I might say it of other diseases also, are really but little affected by medicine, probably because these are not absorbed in sufficient quantity from the digestive tract. Now, what it is desirable to do, is to supply some substance rich in protein, which will be readily assimilated with the least tax on the enfeebled digestive apparatus; and for accomplishing this end, meat juice, eggs, milk, cream and cod liver oil suggest themselves to one's mind, each being valuable articles of nutriment. But as meat juice and milk are not as rich in the desired albuminous principles, in proportion to bulk, as eggs, (a matter of importance as far as the stomach is concerned), and as cream and cod liver oil (being principally fats) not only require considerable expenditure of force in being prepared for assimilation, but are then chiefly valuable in aiding the metamerphosis of nitrogenous matter, rather than as elements of structure, we are bound to give our preference to eggs. Nor will this seem strange when we reflect that nature has supplied in a concentrated form in the egg (which is nearly alike in composition for all animals) all the material necessary for the growth and development of the animal germ.

Perhaps it will not be out of place in this connection to reproduce the following tables, taken from Pavy, showing the composition of the hen's egg.

Analysis of the entire contents :

| | |
|---|---|
| Nitrogenous matter | 14.0 |
| Fatty matter | 10.0 |
| Saline matter | 1.5 |
| Water | 74.0 |

WHITE.

| | |
|---|---|
| Nitrogenous matter | 20.4 |
| Fatty matter | 0.0 |
| Saline matter | 1.6 |
| Water | 78.0 |

YOLK.

| | |
|---|---|
| Nitrogenous matter | 16.0 |
| Fatty matter | 30.7 |
| Saline matter | 1.3 |
| Water | 52.0 |

Thus it will be seen that the white of egg contains no fatty matter, being composed almost wholly of albumen, while the yolk is rich in both nitrogenous and fatty matter. Lehman found in an analysis of thirty (30) eggs, that the albumen averaged about 23.01 grains to each egg. Polecks' analysis is about the same. Lehman also states that he found the fresh white to contain 12.274 per cent. of albumen, and the dried white 92.293 per cent., while the yolk contained not only albumen but fat, phosphates and other salines. Thus I am convinced that eggs are not only richer, in proportion to bulk, in all the proximate principles, than any other article of human food (not even excepting milk, natures nourishment for young mammals), but contain a greater proportion of proetein in the best condition for ready assimilation by a feeble digestive apparatus, and hence; deserve the post of honor in the treatment of Phthisis Pulmonalis and other wasting diseases.

But finding quite often that patients were unable to digest or assimilate the requisite number of eggs, owing perhaps, either to the oil contained, or the inability of the stomach to break up the intimate cell structure, I was led to present them in a dessiccated form, either entire, or just the whites, with to my observation, gratifying results. The mode of preparation is as follows: The eggs are exposed to a warm current of air, never above 110°, F. until they are thoroughly dried, when the residue will be ready for use. As it is more tedious and difficult to evaporate the whole contents, than the whites, I more often throw out the yolk, and afterwards prepare the albumen by adding a little cod liver or other oil; or, triturating it with either egg shell, or one of the phosphates, preferably calcium or sodium phosphate. *Particular attention must* be paid to its preparation, inasmuch as albumen (or dessiccated egg) prepared either by too great heat or by precipitation, whereby it is coagulated, will not yield the same results as that prepared in the manner here given, which requires it readily soluble in warm water, and capable of direct absorption as albuminose by the stomach. I have never observed an instance where it was not borne easily by the stomach when prepared by means of *gentle heat.* It may be administered in teaspoonful doses as a powder with or without one of the salts mentioned, by throwing it in warm water, light soup, or milk, or, emulsified with glycerine or cod liver oil.

Regarding the therapeutic value of eggs, as compared with cod liver oil—*the* standard agent—in the treatment of Pulmonary consumption, it is only necessary to say, that I was at first led to the exclusive use of eggs, by the observation that the administration of the Emul. Ol. Morrh. (made with eggs) produced more positive effects than did the oil alone, and *that* in patients who could equally well take the pure oil. It is unnecessary to add, perhaps, that the eggs must always be fresh and from hens which are properly fed. Albumen made from such eggs in the manner indicated above will keep for a great length of time without undergoing any change.

### ABSTRACTS OF CASES.

I present the following abstracts of my notes of fourteen cases, for the purpose of subjecting to scrutiny the *Diagnosis*, etc., believing, however, that they record unmistakable signs in each, of the earlier stages of the various forms of *Phthisis Pulmonalis*, excepting, perhaps, that variety known as *Acute Tuberculosis.*

Of course I am well aware that some cases of Phthisis are very slow in their march, while others are undoubtedly amenable to cure through nature's unaided efforts, but as instances of the latter fact are comparatively infrequent, the question need not detain us here, neither do I presume to claim for albumen the honor of "a specific" for Phthisis, especially as I have in every instance more or less combined its use with other agents, and hygienic measures hitherto proven to be beneficial in the treatment of this disease. I simply claim that it is superior to cod liver oil, and as previously stated, the *most concentrated* and *powerful* nutriment which can be introduced practically into the wasting economy. And, although by the aid of this agent we may yet be unable to produce a radical cure in every case of incipient "Phthisis," if we can through its instrumentality prolong, for a few years, the life of a victim, its mission ought to be regarded as a valuable one.

1874, Nov. 22d. Case 1.

T. B.—æt. 28. Clerk in R. R. office. Small stature and stooped; small thorax, widower, temperate habits excepting the use of tobacco. Parents American, family history good. Has been ailing more or less for last few years, having had rheumatism, and frequently "colds," especially during *last* year, when the colds have been more obstinate to treatment. Has on two or three occasions, during the past year, expectorated blood in considerable amount also. Now has a "hard cough" with little expectoration, dyspnœa, especially upon even moderate exercise, skin moist usually, but at times dry and feverish, appetite capricious; some indigestion; bowels constipated as a rule.

Physical signs: Percussion sound of upper left side duller than right, auscultation shows crackling and sibilant rale in both infraclavicular regions, more marked in left, and in left mammary region, with broncho-vesicular respiration elsewhere. Pharynx reddened and follicular. Treatment: ℞. Emul. ol. morrh. et albumen. Tablespoonful four times daily. Diet of meat and eggs, etc., and moderate exercise in open air, and to sleep always in an apartment to which fresh air is accessible. It was omitted that a mitral lesion was discovered at the first examination for which XX. gtt. Tr. digital. three times daily was prescribed.

Jan. 8th, 1875. It is noted that the small amount of ol. morrh. which the emulsion contained, apparently disordered his stomach· so that the glycerole of albumen was substituted; and also that the physical signs were rather more unfavorable, being mucous rales and crepitant rales in both upper lobes, with a more decided bronchial character to respiration.

From this time on he improved slowly and quite steadily, notwithstanding, several so-called colds had been contracted, being enabled most of the time to attend to his business duties, and in Nov., 1875, it is noted that he feels better, stronger, and is fleshier than for three years past. During this time the treatment, principally by albumen and eggs, was continued with more or less persistence.

In July, 1876, an exploration of the chest showed an absence of the adventitious sounds previously noted as emanating from the lungs, but a decided *bronchial* character (with short inspiration) to the respiration of the upper part of the left lung. He had "no cough of any amount" nor "spells of fever." Digitalis had been administered more or less for the purpose of controlling the heart's action. He is now (Dec. 8, 1876,) suffering from an attack of acute bronchitis, which is apparently giving way under the use of quinine and ammonia in addition to the other treatment.

### 1875, Jan. 13th. Case II.

Mrs. C. N. B.—æt. 36. Mother of five children who were born at intervals of eighteen months. Short stature, pale and emaciated, with now and then flushed cheeks. Well surrounded with home comforts and necessaries. Has lost two sisters, besides some more distant relatives, with tuberculosis. Parents American, both dead, cause unknown. Has always been feeble, excepting for a month or two at a time. Four months ago she contracted a "cough" which has since been growing in intensity. Menstruation scanty and irregular for past year. Has pain in left side of thorax, cough and expectoration, sometimes mixed with blood, fever and sweating at night. Little appetite, some indigestion, constipation. Says she is rapidly growing weaker, and emaciating.

Physical signs: Percussion sound duller in upper left front. Auscultation shows, a broncho-vesicular character to respiratory murmer, prolonged and jerky expiration with dry crackling in both supra and infra-clavicular region. Upon forced inspiration, subcrepitant and sibilant rales.

Treatment: As she cannot bear cod liver oil well, to take of Emul. albumen et ol. morrh. a tablespoonful four times daily, and spray of carbol. acid (gr. iii. ad. ℥) from steam atomizer. Diet to consist principally of eggs, meat, etc.

Oct. 23. It is noted that she has only been holding her own. Examination revealing no positive improvement of the condition of the lungs. For the last two weeks has been suffering more from indigestion, and great pain of bowels, with diarrhœa at this time. V. gtt. ac. hydrochl. and V. gtt. Tr. nuc. vom. were prescribed to be taken three times daily in water and the Emul. ol. morrh. et. album. was substituted with the glycerole of albumen in tablespoonful doses four times daily, the diet as before to consist largely of milk and eggs. The intestinal derangement was soon relieved and she began to show signs of improvement, and Dec. 17, 1875, it is noted that she has gained considerable in weight and appearance, the cough has greatly diminished, and the physical signs show bronchial respiration in upper left lung especially with duller percussion sound and bronchophony.

She has continued improving up to this writing (Dec., 1876,) with steadiness, only while under the influence of an occasional attack of acute bronchitis, until she is now quite fleshy and feels vigorous.

[TO BE CONCLUDED IN NEXT NUMBER.]

